# Supplementary material for: A Quantitative Assay for Ca2+ Uptake through Normal and Pathological Hemichannels
Source: Int J Mol Sci. 2022 Jun 30;23(13):7337. doi: 10.3390/ijms23137337 (PMC9266989; doi:10.3390/ijms23137337)
Supplement: Supplementary file 1 [file ijms-23-07337-s001.zip › IJMS-1768091- Supplementary Materials.pdf]

Supplementary Materials

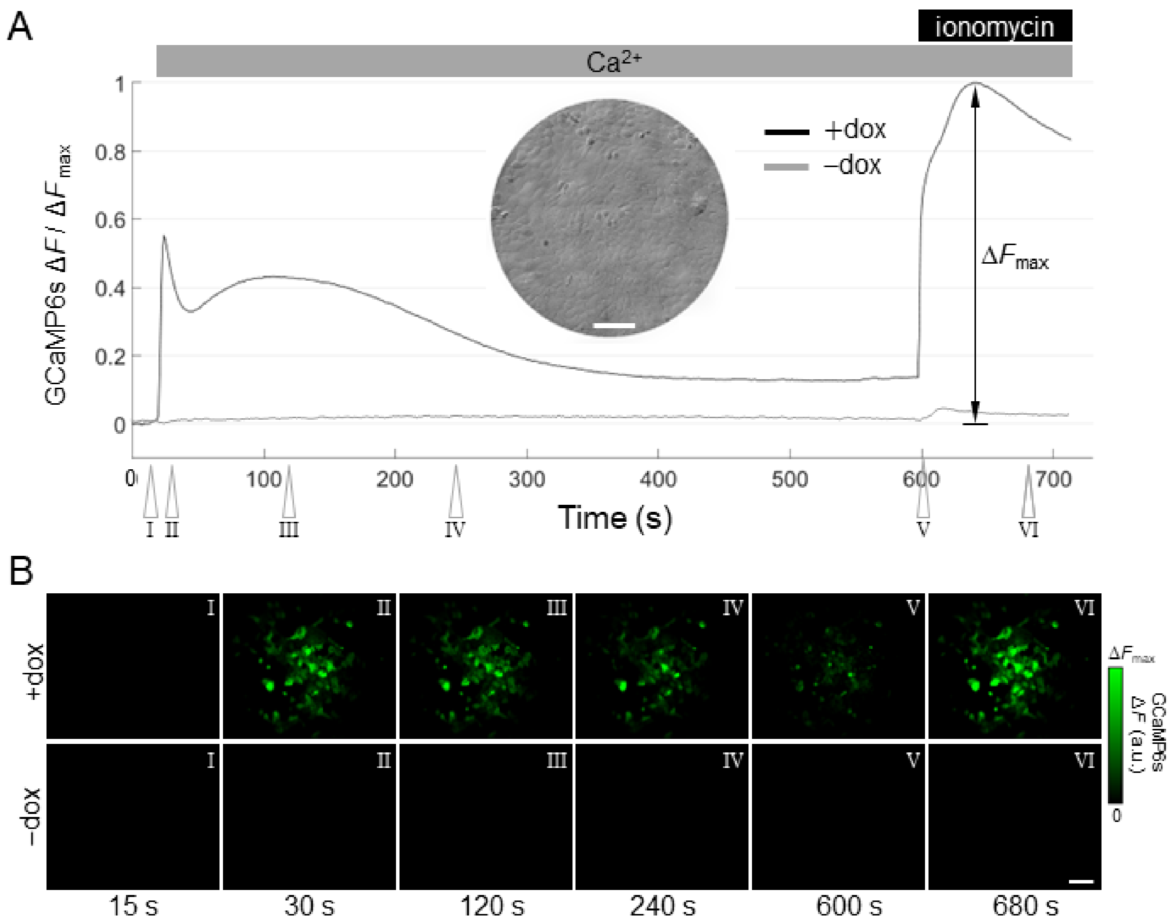

Figure S1. Representative Ca^{2+} uptake experiments in HaCaT-Cx26-GCaMP6s exposed or not exposed to dox. (A) Representative Ca^{2+} uptake experiments performed in cultures exposed (black) or not exposed (grey) to dox (2 μg/mL) for 24 h. $\Delta F/\Delta F_{max}$ traces were obtained by averaging GCaMP6s fluorescence intensity variation, ΔF, in all FOV pixels where baseline intensity exceeded an arbitrary threshold, and normalizing for ΔF_{max}. Time $t = 0$ marks the onset of image acquisition. Inset: representative brightfield image of a cell culture prior to the experiment; scale bar, 100 μm. (B) Representative sequences of fluorescence images acquired at the time points indicated by grey triangles beneath the *x*-axis of the graph in (A) and denoted with matching roman numbers. Scale bar: 100 μm.

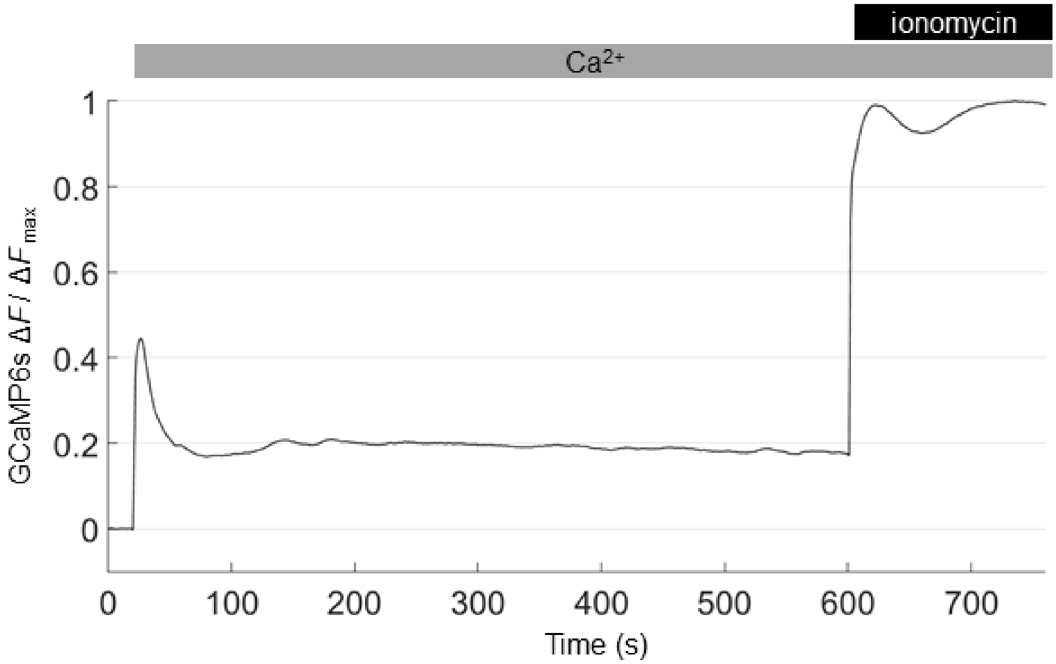

Figure S2. Ca^{2+} uptake response of HaCaT-Cx26-GCaMP6s cells exposed to an initial $[Ca^{2+}]_{ex}$ of 20 μM. Representative $\Delta F/\Delta F_{max}$ trace obtained from a HaCaT-Cx26-GCaMP6s cell culture kept in a low initial $[Ca^{2+}]_{ex}$ (20 μM) and then exposed to a 1 M $CaCl_2$ bolus (t = 20 s). Time t = 0 marks the onset of image acquisition.

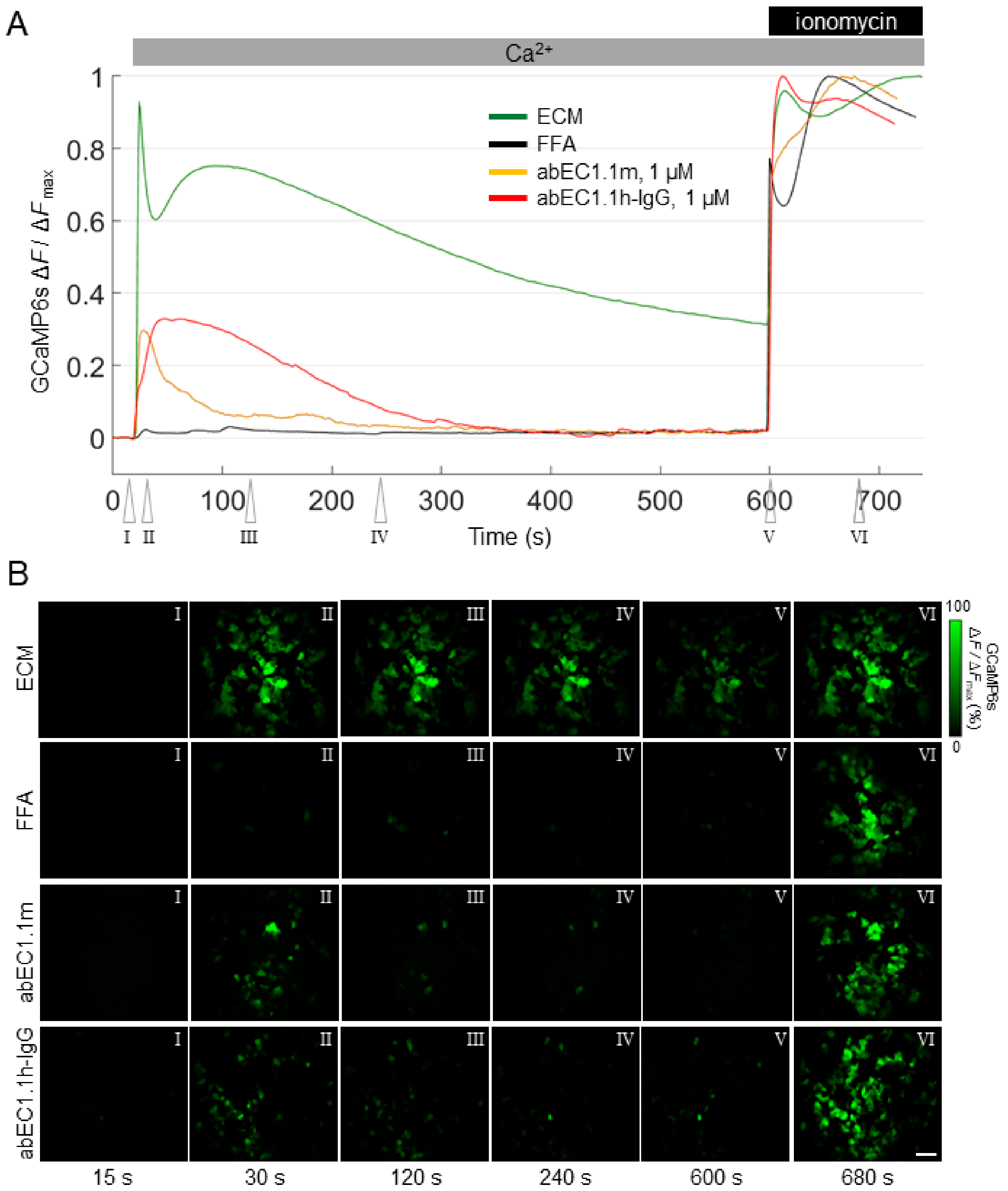

Figure S3. Representative Ca^{2+} uptake experiments for conditions shown in Figure 4C,D. (A) $\Delta F/\Delta F_{max}$ traces obtained from four HaCaT-Cx26-GCaMP6s cell cultures in one of the following extracellular media: ECM (green), ECM plus FFA (100 µM, black), ECM plus abEC1.1m (1 µM, orange) and ECM plus abEC1.1h-IgG (1 µM, red). Time $t = 0$ marks the onset of image acquisition. (B) Corresponding sequences of fluorescence images acquired at the time points indicated by gray triangles beneath the x-axis of the graph in (A) and denoted with matching roman numbers. Scale bar: 100 µm.

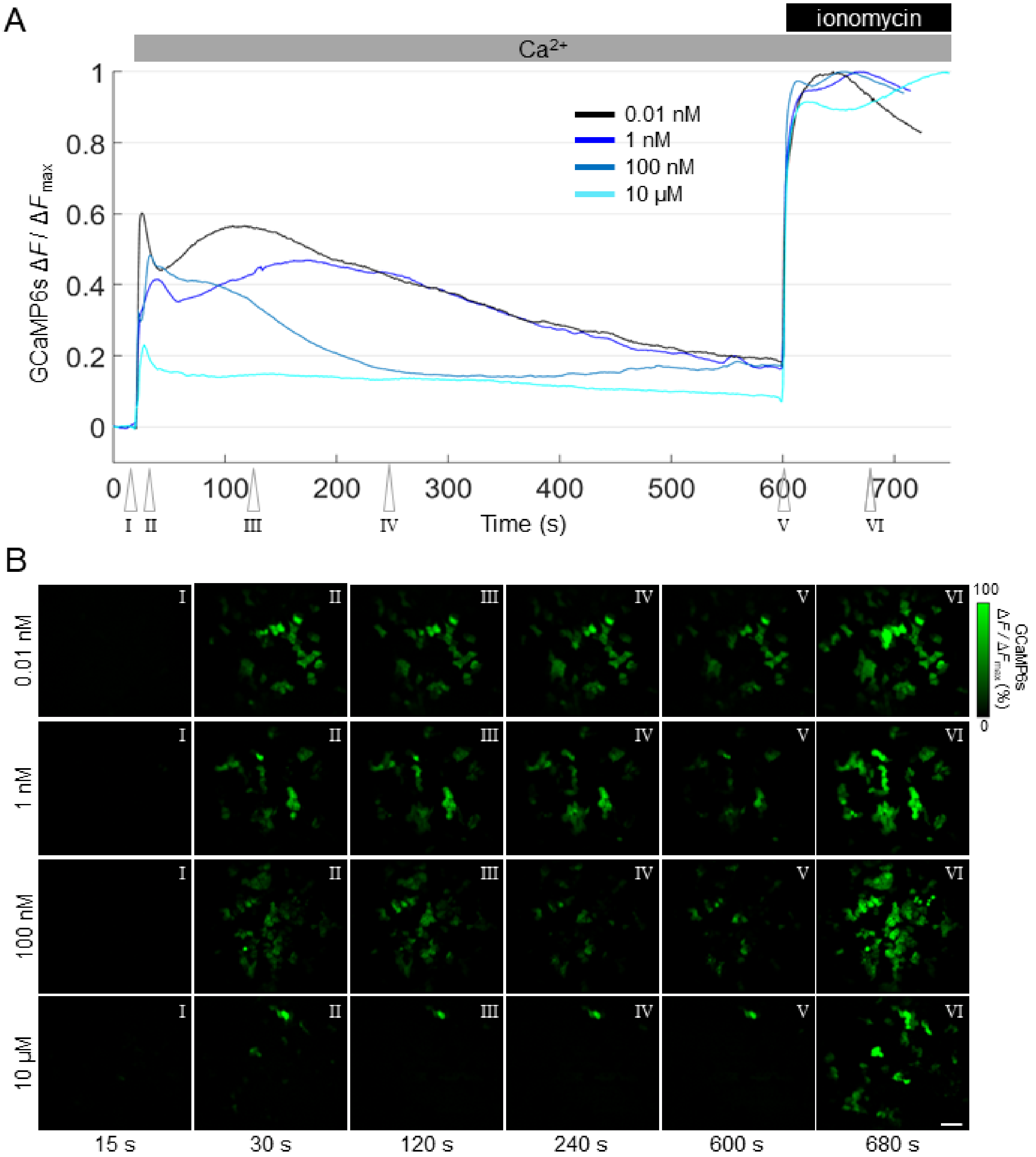

Figure S4. Representative Ca^{2+} uptake experiments for conditions shown in Figure 4E. (A) $\Delta F/\Delta F_{max}$ traces obtained from four HaCaT-Cx26-GCaMP6s cell cultures in ECM supplemented by abEC1.1h-IgG at one of the following concentrations: 0.01 nM (black), 1 nM (dark blue), 100 nM (light blue) and 10 µM (cyan). Time $t = 0$ marks the onset of image acquisition. (B) Corresponding sequences of fluorescence images acquired at the time points indicated by gray triangles beneath the x-axis of the graph in (A) and denoted with matching roman numbers. Scale bar: 100 µm.
